# The Effect of Preselection on the Level of Bias and Accuracy in a Broiler Breeder Population, a Simulation Study

**DOI:** 10.1111/jbg.12908

**Published:** 2024-11-21

**Authors:** Charlie A. de Hollander, Thinh T. Chu, Danye Marois, Vivian B. Felipe, Fernando B. Lopes, Mario P. L. Calus

**Affiliations:** ^1^ Cobb Vantress Inc Siloam Springs Arkansas USA; ^2^ Animal Breeding and Genomics Wageningen University and Research Wageningen The Netherlands; ^3^ Department of Molecular Biology and Genetics, Center for Quantitative Genetics and Genomics Aarhus University Tjele Denmark

**Keywords:** accuracy, bias, broiler breeding, preselection

## Abstract

Many breeding programmes have to perform preselection, as genotyping and phenotyping all potential breeder candidates is often not a feasible option. There is need to understand how preselection affects the quality of the genomic estimated breeding values (EBVs) at final selection and thereby can affect genetic progress. This simulation study evaluated nine different preselection strategies in a broiler breeder programme and their effect on the quality of the (genomic) EBVs and genetic progress for three different traits: body weight (Body Weight), residual feed intake (RFI) and body weight gain (Gain). All birds have Body Weight recorded at preselection, but only the preselected birds were phenotyped for RFI and Gain and genotyped. The following criteria and intensities were studied: preselection based on phenotypic Body Weight (P), on a BLUP index (B) or on an ssGBLUP Index (G). Additionally, all criteria were studied with three different selection intensities, 10% of the males and 30% of the females (P10, B10, G10), 15% of the males and 45% of the females (P15, B15, G15) and 20% of the males and 60% of the females (P20, B20, G20). The accuracy at preselection with G10 was more accurate than B10 for both RFI and Gain (0.71 vs. 0.58 and 0.65 vs. 0.55 respectively), and also G15 was more accurate than B15 for both RFI and Gain (0.72 vs. 0.63 and 0.67 vs. 0.64 respectively); thus, the difference in accuracy reduces with an increasing number of birds being preselected. Differences in accuracy at final selection were mostly notable in the RFI trait between P10, B10 and G10, where G10 showed the highest accuracy (0.82 vs. 0.84 vs. 0.86 respectively). This difference in accuracy for RFI disappeared when more animals were preselected. For Body Weight and Gain, the BLUP preselection resulted in the highest accuracy at final selection when selection intensity decreased. The dispersion bias of EBVs at final selection was most pronounced in the P10 and P15 for Body Weight (0.81 and 0.92) but disappeared at P20 (0.97). The dispersion bias for all other criteria and traits was relatively small. Genetic progress was mostly affected when P10 or P15 was used at preselection, where the progress in Body Weight was noticeably higher, but prominently lower for RFI and Gain. The BLUP and ssGBLUP preselection had very similar genetic progress across traits and showed comparable improvements in the selection index. In conclusion, with high preselection intensity, the use of ssGBLUP at preselection might be favoured as there is an improvement in genetic progress across traits in all scenarios, which is due to the increased preselection accuracy. When preselection intensity decreases, the benefit of using ssGBLUP over BLUP at preselection disappears.

## 1 Introduction

Breeding companies aim to maximise genetic progress for economically important traits while minimising the costs. It is important to keep optimising breeding strategies as the breeding programme and associated technology evolve. Genetic progress depends on four main components: selection intensity, genetic variance, generation interval and accuracy of selection. Since genetic progress is cumulative, small improvements in these components will have a significant benefit over time. Selection accuracy is directly impacted by the accuracy of estimated breeding values (EBVs). If accuracy is high, a breeding company is better able to identify the animals with the best genetic merit to become breeders and thereby increase their yearly genetic progress.

The quality of EBVs depends on correct collection of pedigree, phenotype and genotype information. In a perfect setting, all animals in a breeding programme would be phenotyped and genotyped. However, due to logistical and financial constraints, this is not always feasible. Therefore, selective phenotyping and/or genotyping is common practice in most species. To do this optimally, the amount of information collected and what individuals should be represented needs to be evaluated. Preselection results in phenotypic and/or genomic data being available on a non‐random pool of animals, which may jeopardise the normal distribution of specific data characteristics and can potentially lead to bias and lowered accuracy of the (Genomic) EBVs ((G)EBVs) (Appel et al. 1998; Ehsani, Janss, and Christensen 2010; Howard et al. 2018; Jibrila et al. 2021; Pollak, Van der Werf, and Quaas 1984; Schisterman et al. 2013; Vitezica et al. 2011; Zhao et al. 2012). Preselection based on phenotypes or a selection index implies that the assumption of random sampling in an animal model does not hold (Henderson 1975; Henderson et al. 1959). In other words, the preselection changes the null expectation of the Mendelian Sampling within families, which may result in bias of the (G)EBVs (Appel et al. 1998; Ehsani, Janss, and Christensen 2010; Howard et al. 2018; Jiménez‐Montero, Gonzalez‐Recio, and Alenda 2012; Patry and Ducrocq 2011; Pollak, Van der Werf, and Quaas 1984; Schaeffer, Schenkel, and Fries 1998; Sullivan 2019). In dairy cattle, bias caused by intensive genomic preselection of bulls has been extensively explored (Patry and Ducrocq 2011; Patry, Jorjani, and Ducrocq 2013; Togashi et al. 2018; VanRaden et al. 2009; Vitezica et al. 2011). The higher the preselection intensity, the larger the bias of the GEBVs, which is due to the fact that there is an increase in non‐randomly missing data (Patry and Ducrocq 2011). A proposed solution is to use the Single Step Genomic BLUP (ssGBLUP) model, as it combines the genomic relationships from genotyped individuals with pedigree relationships of all individuals that are not genotyped and thereby allows for data of unselected non‐genotyped animals to be included (Christensen and Lund 2010; Legarra, Aguilar, and Misztal 2009; Misztal, Legarra, and Aguilar 2009). It was found in dairy cattle that bias indeed could be reduced by using ssGBLUP (Legarra et al. 2014; Misztal, Legarra, and Aguilar 2009; Vitezica et al. 2011).

In a broiler breeder company, there are thousands of chicks hatched every week and preselection is implemented to determine which chicks will be phenotyped for subsequent traits and/or genotyped. Preselection decisions are often based on selecting the best performing animals or those with the highest index value, with the aim to select animals with the best genetic potential for traits of interest to become breeders. However, the impact of these preselection decisions in terms of bias and accuracy of GEBVs use for final selection and thereby its impact on the genetic progress is not well known.

The traits of interest in this study are body weight (Body Weight), residual feed intake (RFI) and body weight gain (Gain), as these are important broiler performance traits. Different preselection criteria will lead to different animals being phenotyped and genotyped and to be available at the final selection stage. Thus, the objectives of this study were to evaluate (1) the effect of preselection intensity on the bias and accuracy of GEBVs at final selection, (2) the effect of different preselection criteria on the bias and accuracy of GEBVs at final selection and (3) identify which combination of selection intensity and criteria optimise the rate of genetic progress for traits of interest for a broiler breeder company.

## 2 Material and Methods

To identify the best preselection strategy for a broiler breeding company, there is need to compare multiple scenarios with different preselection strategies. This was done by simulating the same breeding scheme but applying different selection intensities and criteria for preselection. Afterwards, the accuracy and bias of the GEBVs used for final selection and the achieved genetic progress were compared for all different preselection strategies for Body Weight, RFI and Gain.

### 2.1 Simulated Data

The breeding population was simulated in three steps. In the first and second step, the historical and founding population were generated by the use of the stochastic simulation program QMSIM (Sargolzaei and Schenkel 2009), while in the third step, the breeding scheme was simulated by the simulation program ADAM (Pedersen et al. 2009). This simulation strategy followed previous studies (Chu et al. 2018) to take advantage of the efficiency of the QMSIM software for the first two steps, while taking advantage of the functionality and flexibility of ADAM to simulate a more detailed multi‐trait breeding programme in the third step, which is not possible with QM‐SIM. The simulated genome consisted of 28 chromosomes, with lengths ranging between 45 and 484 cM with a total of 2930 cM, based on the lengths of the chromosomes across the chicken genome (Groenen et al. 2009).

**FIGURE 1:**
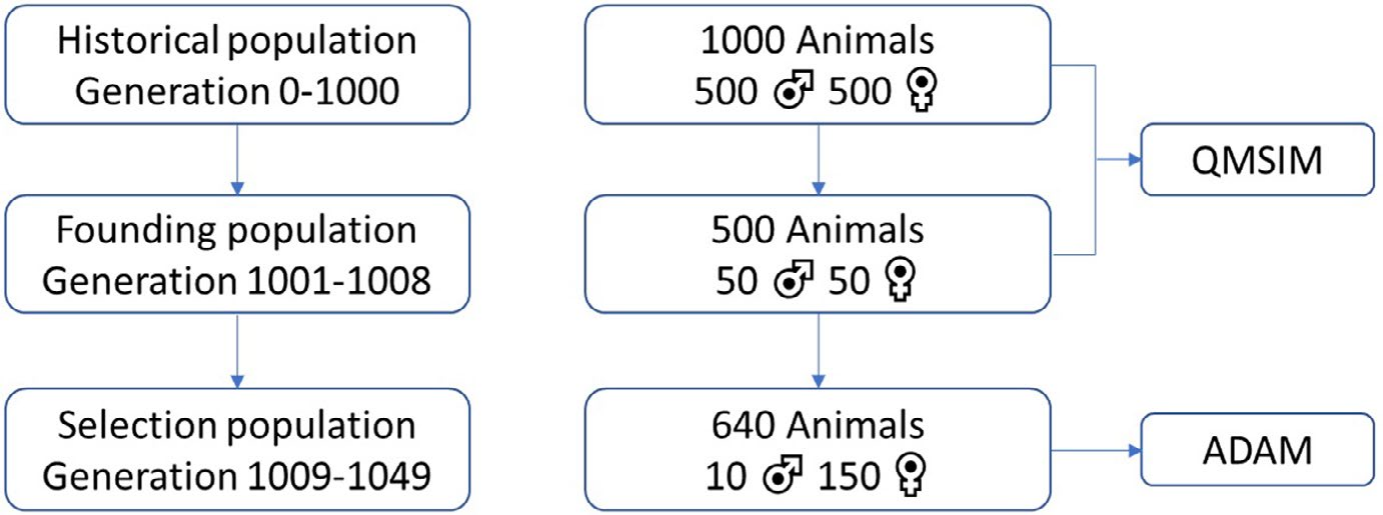
An overview of the generation of the historical and base population that is used in the breeding scheme. [Colour figure can be viewed at wileyonlinelibrary.com]

The historical population was simulated for 1000 generations to establish mutation‐drift equilibrium and linkage disequilibrium between loci. The population had a consistent population size of 1000 birds per generation, with equal numbers of individuals from both sexes, random mating and no selection or migration. Loci had two alleles with equal frequencies in the first historical generation, and a recurrent mutation rate of $2.5\times 10^{-8}$ thereafter. In the second step, the founding population was created. From the last generation of the historical population, 50 founder females and 50 founder males were randomly selected and were randomly mated, and each mating generated 10 offspring. This was done for another seven generations, and the last generation was used as founders for the next step. Until this point, no phenotypes were simulated. From all the segregating loci with a minor allele frequency of at least 0.05, 52 k and 3 k loci were randomly selected, respectively, to be markers and quantitative trait loci (QTL). From the founders, a base population was created by randomly taking haplotypes from two random founders to create 320 males and 4800 females. From these base individuals, 10 males and 150 females were chosen randomly to be parents in the early time steps, until the selected birds from the first time steps were sufficiently mature to be parents. This base population was used in the simulation of the breeding scheme simulated by ADAM, and at this moment, the simulation of phenotypes started. An overview of the simulations and populations is depicted in Figure 1.

### 2.2 Breeding Scheme

**FIGURE 2:**
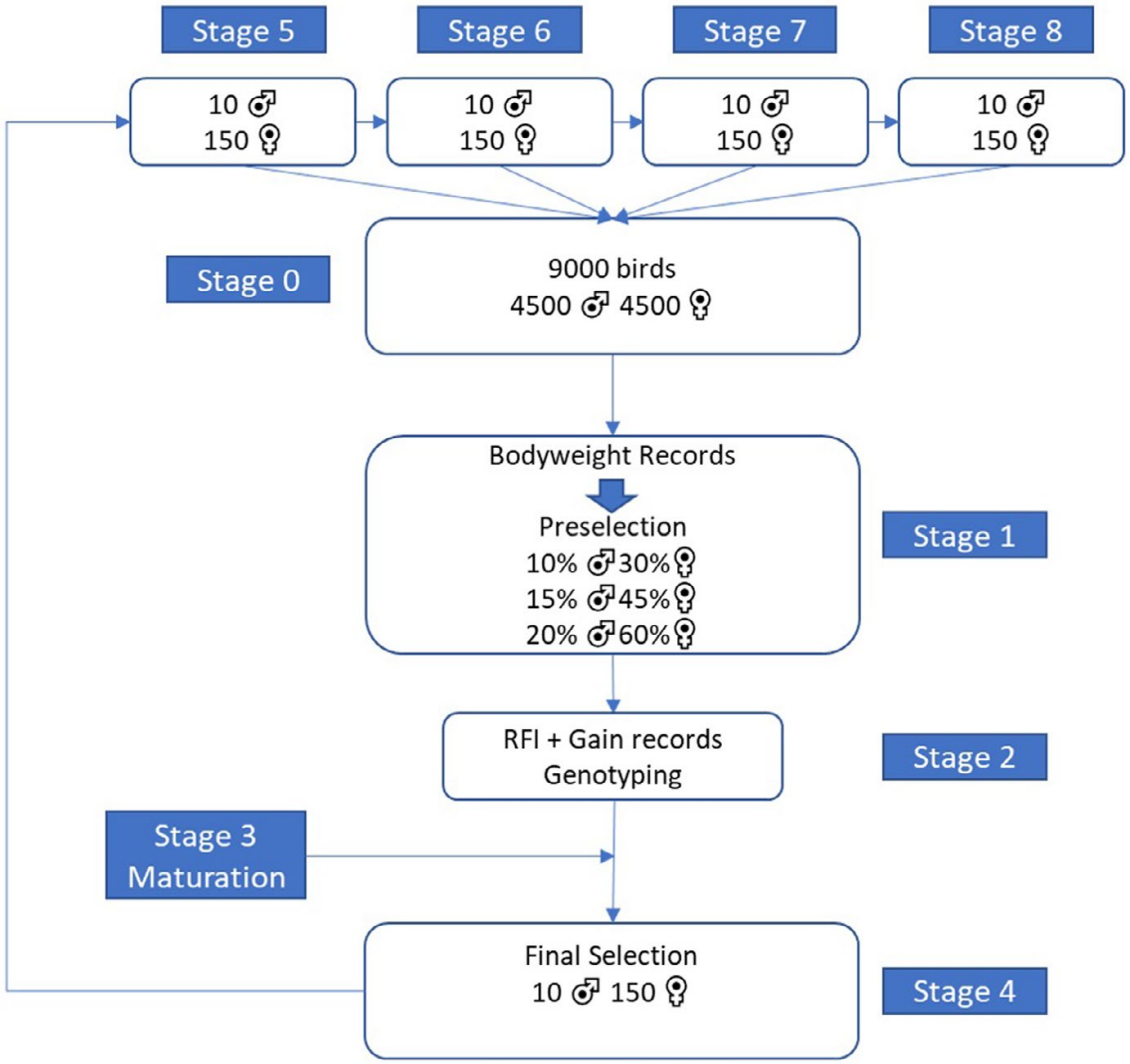
The broiler breeding scheme simulated in step 3. [Colour figure can be viewed at wileyonlinelibrary.com]

The breeding scheme was simulated by ADAM. For each breeding scheme, 40 selection rounds were performed and each selection round consisted of eight stages (see Figure 2 for an overview). Each selection round started at stage 0 with 9000 chicks that hatched with a 50:50 sex ratio. In stage 1, the Body Weight of all birds was recorded, and based on the selection strategy, different proportions of birds were preselected based on either their phenotype or their selection index. In stage 2, the preselected birds were phenotyped for RFI and Gain and genotyped. In stage 3, the birds grew to maturity. In stage 4, the second and final selection took place, where 10 males and 150 females from the preselected birds were selected based on the selection index (as described later) to become breeder candidates and produce the next generation for the breeding scheme. After the final selection, every male was mated to 15 females and their reproductive stages were stage 5–8. After completing stage 8, the birds were removed from the breeding program. In every reproductive stage, the breeders produced chicks, however, when a breeder got older, the number of chicks per reproductive stage declined. In a selection round, which consists of seven hatches, and each hatch consists on average of 1286 chicks, the female breeders hatched 16 chicks at stage 5 and 6, 15 chicks at stage 7 and 13 chicks at stage 8. Thus, in total, each female breeder hatched 60 chicks and each male breeder hatched 900 chicks in their reproductive cycle.

### 2.3 Trait Simulation

The traits for the breeding scheme were simulated with the simulation program ADAM. In our simulated breeding scheme, there are three traits considered in the breeding goal, body weight, Residual Feed Intake (RFI) and gain. The phenotypes $\left(y\right)$ of Body Weight were simulated as $y_{\mathrm{ji}}=x_{i}+b_{j}+g_{i}+c_{i}+e_{i}$ and for RFI and Gain as: $y_{\mathrm{ji}}=x_{i}+b_{j}+g_{i}+e_{i}$, where $y_{\mathrm{ji}}$ is the phenotype for individual *i* born in hatch *j*, $x_{i}$ is the contemporary group effect (defined for each parents age group × age group × gender group), $b_{j}$ is the effect for hatch *j*, $g_{i}$ is the true breeding value (TBV), $c_{i}$ is the maternal permanent environmental effect and $e_{i}$ is an environmental effect for the *i*th bird. Hatch effects were drawn from a normal distribution with mean 0 and a variance that ranged between 1% and 3% of the phenotypic variance of the traits. Variances and covariances of the simulated random effects were based on variance components estimated from real data for the three traits (Table 1). Each bird that was hatched in the simulation had TBVs for all three traits. TBVs were calculated based on the individual genotypes of all 3000 QTLs and their simulated effects. The simulated QTL effects followed the variances and genetic correlations in Table 1, such that those were also achieved between the TBV of the three traits. To this end, the effect of each QTL was drawn from a normal distribution *N*(0,1), and then centred and rescaled to achieve the following $\mathrm{MVN}\left(\left[\begin{array}{c} 0\\ 0\\ 0 \end{array}\right],\left[\begin{array}{ccc} h_{\mathrm{BW}}^{2} & \sigma_{\mathrm{gBW},\mathrm{RFI}} & \sigma_{\mathrm{gBW},G}\\ \sigma_{\text{gRFI},\mathrm{BW}} & h_{\mathrm{RFI}}^{2} & \sigma_{\text{gRFI},G}\\ \sigma_{\mathrm{gG},\mathrm{BW}} & \sigma_{\mathrm{gG},\mathrm{RFI}} & h_{G}^{2} \end{array}\right]\right)$, where $h_{\mathrm{BW}}^{2},$ $h_{\mathrm{RFI}}^{2}$ and $h_{G}^{2}$ are, respectively, the heritabilities of Body Weight, RFI and Gain. Note that we effectively assumed that the phenotypic variance for each trait was equal to 1, and therefore, the genetic variance for each trait was equal to its heritability. Thus, the genetic covariances among the traits, $\sigma_{\mathrm{gBW},\mathrm{RFI}}$, $\sigma_{\mathrm{gBW},G}$ and $\sigma_{\text{gRFI},G}$ were computed as, for example, $\sigma_{\mathrm{gBW},\mathrm{RFI}}=r_{\mathrm{gBW},\mathrm{RFI}}\sqrt{h_{\mathrm{BW}}^{2}h_{\mathrm{RFI}}^{2}}$ using the genetic correlations among the traits ($r_{\mathrm{gBW},\mathrm{RFI}}$, $r_{\mathrm{gBW},G}$ and $r_{\text{gRFI},G}$) as shown in Table 1. These QTL effects are then kept constant across the breeding scheme for calculating TBV of individual birds based on their QTL genotypes. Maternal permanent environmental effects for Body Weight were drawn from $N\left(0,c^{2}\right)$, where $c^{2}$ is the proportion of variance explained by the maternal permanent environmental effect, which had a value of 0.04. The environmental effects were drawn from $\mathrm{MVN}\left(\left[\begin{array}{c} 0\\ 0\\ 0 \end{array}\right],\left[\begin{array}{ccc} 1-h_{\mathrm{BW}}^{2}-c^{2} & \sigma_{\mathrm{eBW},\mathrm{RFI}} & \sigma_{\mathrm{eBW},G}\\ \sigma_{\text{eRFI},\mathrm{BW}} & 1-h_{\mathrm{RFI}}^{2} & \sigma_{\text{eRFI},G}\\ \sigma_{\mathrm{eG},\mathrm{BW}} & \sigma_{\mathrm{eG},\mathrm{RFI}} & {1-h}_{G}^{2} \end{array}\right]\right)$ where $\sigma_{\mathrm{eBW},\mathrm{RFI}}$, $\sigma_{\mathrm{eBW},G}$ and $\sigma_{\text{eRFI},G}$ are the residual covariances among the traits that are computed as, for example, $\sigma_{\mathrm{eBW},\mathrm{RFI}}=r_{\mathrm{eBW},\mathrm{RFI}}\sqrt{\left(1-h_{\mathrm{BW}}^{2}-c^{2}\right)\left(1-h_{\mathrm{RFI}}^{2}\right)}$ using the residual correlations among the traits ($r_{\mathrm{eBW},\mathrm{RFI}}$, $r_{\mathrm{eBW},G}$, and $r_{\text{eRFI},G}$) as shown in Table 1.

**TABLE 1:** Heritabilities (diagonal), genetic correlations (below the diagonal) and residual correlations (above the diagonal).

	Body weight	RFI	Gain
Body weight	0.18	−0.08	0.07
RFI	0.11	0.19	−0.04
Gain	0.15	0.14	0.17

### 2.4 Preselection Scenarios

We considered three breeding schemes that were different with respect to the preselection applied to the youngest generation of animals. Every breeding scheme simulated a different preselection strategy consisting of three different preselection criteria and intensities. Each breeding scheme was ran for 40 selection rounds and replicated 15 times to establish consistent estimates. At preselection, animals only had body weight recorded on them, while preselected birds were thereafter phenotyped for RFI and gain and genotyped. Thus, at the time of preselection, there were no phenotypes for RFI and gain and no genotypes available for the birds themselves.

The following preselection criteria were simulated:

1. Preselection based on phenotypes for body weight (P), the heaviest chicks were selected to become breeder candidates
2. Preselection based on an index calculated from BLUP EBVs (B), the chicks with the highest selection index were selected to become breeder candidates
3. Preselection based on index calculated from ssGBLUP GEBVs (G), the chicks with the highest selection index were selected to become breeder candidates

The following standardised index was used for preselection based on selection criteria 1 and 2:

$$H=1\times \text{Weight}\;\left(G\right)\mathrm{EBV}-0.82\times \mathrm{FE}\;\left(G\right)\mathrm{EBV}+0.50\times \text{Gain}\;\left(G\right)\mathrm{EBV}.$$

where (G)EBVs are expressed in genetic standard deviation units, and the index weights are expressed per genetic standard deviation unit of each of the traits, and scaled such that the index weight for Body Weight was 1. Finally, it should be noted that with preselection based on ssGBLUP GEBVs, the genotypes of the parents are used to compute the GEBVs of the breeder candidates, while the breeder candidates themselves do not yet have genotypes available, as genotyping only took place after preselection.

The following selection intensities were simulated for each of the three criteria:

1. 10% males (450) and 30% females (1350) based on phenotype (P10), BLUP EBVs (B10) or ssGBLUP EBVs (G10)
2. 15% (675) males and 45% females (2025) based on phenotype (P15), BLUP EBVs (B15) or ssGBLUP EBVs (G15)
3. 20% (900) males and 60% females (2700) based on phenotype (P20), BLUP EBVs (B20) or ssGBLUP EBVs (G20)

The number of chicks phenotyped for each of the traits, as well as the number of chicks genotyped, is presented for each preselection strategy in Table 2. Following each of the preselection strategies, final selection to select breeders was always based on the same selection index as used for preselection, and this selection index was computed from GEBV obtained with ssGBLUP. Across all strategies and replicates, on average 51,525 segregating markers, with an MAF higher than 0.05, were used for the ssGBLUP analyses at preselection and final selection.

**TABLE 2:** Number of phenotypes and genotypes for each preselection strategy.

	Number of phenotypes body weight		Number of phenotypes RFI and gain		Number of genotypes	
Strategy^a^	♂	♀	♂	♀	♂	♀
P10	4500	4500	450	1350	450	1350
B10	4500	4500	450	1350	450	1350
G10	4500	4500	450	1350	450	1350
P15	4500	4500	675	2025	675	2025
B15	4500	4500	675	2025	675	2025
G15	4500	4500	675	2025	675	2025
P20	4500	4500	900	2700	900	2700
B20	4500	4500	900	2700	900	2700
G20	4500	4500	900	2700	900	2700

^a^ P: phenotype (body weight); B: pedigree‐based BLUP; G: single‐step genomic BLUP.

### 2.5 Models

Estimated breeding values were based on the following multivariate ssGBLUP model:

y=Xb+Zg+Wc+e

where y is the vector of the phenotypic records for Body Weight, Gain and RFI; b is a vector of fixed effects of hatches; g is a vector of breeding values for the traits Body Weight, RFI and Gain assumed to be normally distributed as *MVN* $\left[\begin{array}{c} 0\\ 0\\ 0 \end{array},H\;⨂\;V_{g}\right]$, where **H** is a combined pedigree and genomic relationship matrix, as explained in more detail below. In all analyses, for the variances we used the simulated values as defined in the above. Thus, the matrix $V_{g}=\left[\begin{array}{ccc} h_{\mathrm{BW}}^{2} & \sigma_{\mathrm{gBW},\mathrm{RFI}} & \sigma_{\mathrm{gBW},G}\\ \sigma_{\text{gRFI},\mathrm{BW}} & h_{\mathrm{RFI}}^{2} & \sigma_{\text{gRFI},G}\\ \sigma_{\mathrm{gG},\mathrm{BW}} & \sigma_{\mathrm{gG},\mathrm{RFI}} & h_{G}^{2} \end{array}\right]$ is the 3X3 covariance matrix of Body Weight, RFI and Gain; ⨂ is the Kronecker product; and c is the vector of permanent environmental effects assumed to be normally distributed as $N\left(0,Ic^{2}\right)$. This permanent environment effect is only included in the model for Body Weight, but not for the traits of RFI and Gain. The X, Z and W are incidence matrices.

Finally, the vector of random residuals e was assumed to follow a *MVN* $\left[\begin{array}{c} 0\\ 0\\ 0 \end{array},I⨂R\right]$

Where $R=\left[\begin{array}{ccc} 1-h_{\mathrm{BW}}^{2}-c^{2} & \sigma_{\mathrm{eBW},\mathrm{RFI}} & \sigma_{\mathrm{eBW},G}\\ \sigma_{\text{eRFI},\mathrm{BW}} & 1-h_{\mathrm{RFI}}^{2} & \sigma_{\text{eRFI},G}\\ \sigma_{\mathrm{eG},\mathrm{BW}} & \sigma_{\mathrm{eG},\mathrm{RFI}} & {1-h}_{G}^{2} \end{array}\right]$ and I is an identity matrix.

In the ssGBLUP models, the inverse of the H matrix, $H^{-1}$, was constructed (Aguilar et al. 2011; Christensen and Lund 2010) combining $A^{-1}$, the inverse of the pedigree relationship matrix of the genotyped animals ($A_{22}^{-1}$) and the inverse of the genomic relationship matrix $G^{*}$. The matrix $G^{*}$ was computed as $G^{*}=0.95G+0.05A_{22}$, where G was based on the first method described by VanRaden (2008) using allele frequencies in the base population of the simulation, to make G and $A_{22}$ compatible. Inbreeding was included in the computation of the matrices $A^{-1}$ and $A_{22}^{-1}$.

The BLUP model was similar as the ssGBLUP model, except that breeding values were assumed to be normally distributed as MVN $\left[\begin{array}{c} 0\\ 0\\ 0 \end{array},\begin{array}{ccc} A & ⨂ & V_{g} \end{array}\right]$, where **A** is the matrix of additive genetic relationships computed from pedigree data, instead of using **H** as in the ssGBLUP model. The pedigree was traced back to the base population.

The (G)EBV was calculated for all hatched individuals at preselection, and subsequently GEBVs were estimated for all preselected individuals present at final selection. Information on all individuals placed at hatch was used in the estimation of GEBVs at final selection. Both BLUP and ssGBLUP models used true variance components for prediction of breeding values. The values for these true variance components were based on estimates from a large data set from a real pure line population from Cobb Vantress Inc. (Siloam Springs, Arkansas, USA). Computations were carried out using the DMU5 model of the DMU package (Madsen and Jensen 2013). The estimation in time step 1 and 4 used all the information available since selection round 1.

### 2.6 Evaluated Parameters

#### 2.6.1 Accuracy $\left(r\right)$ at Preselection and Final Selection

The accuracy of preselection was defined for the BLUP and ssGBLUP preselection strategies, respectively, as the correlation between TBV and EBV (BLUP) or GEBV (ssGBLUP) at preselection, for all breeder candidates available at preselection in one selection round. The accuracy at final selection was defined as the correlation between the TBV and GEBV values of all breeder candidates available at final selection of one selection round. Both accuracies were calculated per trait for each preselection strategy and averaged over the last 11 selection rounds (30–40) and over the 15 replicates for each simulation. The last 11 selection rounds were evaluated to derive the main results as those showed a consistent and stable improvement of TBVs for body weight, RFI and gain and consistent accuracies.

#### 2.6.2 Family Representation at Preselection and Final Selection

The family representation is evaluated for all preselection criteria and intensities at both the preselection and final selection stage. The representation is expressed as the number of dam families of which candidates were selected.

#### 2.6.3 Magnitude of Dispersion Bias of GEBVs at Final Selection

The magnitude of the dispersion bias was assessed by calculating the regression coefficient $\left(\mathrm{RC}\right)$ of the TBVs on the GEBVs at final selection for all available breeder candidates within each selection round. The presented bias are the RCs averaged over the last 11 selection rounds of each simulation, and then averaged over all 15 replicates.

#### 2.6.4 Standard Error (SE)

The standard errors for the accuracy and RC between the TBV and GEBVs across the simulations were calculated using the following formula:

$$\mathrm{SE}=\frac{\sigma }{\sqrt{n}}$$

where σ is the standard deviation of RC or *r* across replicates, and *n* is the number of replicates.

#### 2.6.5 Genetic Progress

The genetic progress for each trait was standardised by dividing the progress by its additive genetic standard deviation and is expressed in genetic standard deviations (Genetic SD). This was done for both the TBVs and GBVs to compare true and estimated genetic progress. The standardised genetic progress is calculated as the difference between the average TBVs or GEBVs of the available breeder candidates at final selection between selection rounds 30 and 40. The computed genetic progress was averaged over the 15 replicates per strategy and calculated only for the breeder candidates present at final selection. Additionally, this study calculated the differences in progress for the selection index between each selection criteria. This was also done between selection rounds 30 and 40 and averaged over all 15 replicates. A *t*‐test was performed to evaluate if the genetic progress in each trait by selection intensity combination was significantly different between preselection strategies.

## 3 Results

Across all simulated loci, minor allele frequencies were uniformly distributed for both SNPs and QTLs (results not shown). The LD pattern between loci observed in the founder haplotypes is presented in Figure S1. Assuming roughly that 1 Mb corresponds to 2.5 cM for the largest 10 chromosomes (Groenen et al. 2009), this pattern agrees closely with LD patterns observed in broiler chickens in practice (Fu et al. 2015). Below we describe the results for all three different preselection criteria (P, B and G) and three different selection criteria for males and females 10:30% (10), 15:45% (15) and 20:60% (20).

### 3.1 Preselection Accuracy

The accuracy of both BLUP and ssGBLUP preselection is shown in Table 3. There is clear benefit of using ssGBLUP instead of BLUP for preselection for all preselection intensities, as the accuracies are higher for all traits. Especially comparing B10 to G10 for Body Weight (0.63 and 0.68 respectively), RFI (0.58 and 0.71 respectively) and gain (0.55 and 0.65 respectively). For B15 and G15, the advantage for RFI and gain is smaller but still present, whereas the benefit of ssGBLUP for body weight is large across all intensities. When the preselection intensity reaches 20:60%, BLUP is still being outperformed by ssGBLUP for body weight (0.63 and 0.71 respectively) and RFI (0.63 and 0.69 respectively) but wins unexpectedly for gain (0.69 and 0.64 respectively). With more animals being preselected, the accuracy increases for both BLUP and ssGBLUP for RFI and Gain. These improvements are very minimal for body weight, and surprisingly, B15 actually shows lower accuracy compared to B10 (0.59 and 0.63 respectively).

**TABLE 3:** Preselection GEBV accuracy per trait for each preselection scenario averaged over the last 11 selection rounds and the 15 replicates.

Criteria	Preselection % ♂♀	Abbreviation	Body weight		RFI		Gain	
			Accuracy	SE	Accuracy	SE	Accuracy	SE
BLUP	10:30	B10	0.63	0.003	0.58	0.003	0.55	0.005
ssGBLUP	10:30	G10	0.68	0.003	0.71	0.003	0.65	0.002
BLUP	15:45	B15	0.59	0.004	0.63	0.003	0.64	0.003
ssGBLUP	15:45	G15	0.70	0.003	0.72	0.004	0.67	0.005
BLUP	20:60	B20	0.63	0.003	0.63	0.002	0.69	0.005
ssGBLUP	20:60	G20	0.71	0.002	0.69	0.004	0.64	0.002

### 3.2 Final Selection Accuracy

The obtained GEBV accuracy at final selection for each trait, preselection intensity and criteria is shown in Table 4. The obtained final accuracy at a 10:30% preselection intensity is similar when preselection is based on phenotypes, BLUP or ssGBLUP for body weight (~0.80) and gain (~0.81), while for RFI, the accuracy stepwise increases going from using phenotypes to BLUP to ssGBLUP (0.82, 0.84 and 0.86 respectively). At a 15:45% preselection intensity, across all selection criteria, similar accuracies were obtained for body weight. Accuracy of GEBVs for RFI and gain were greater following preselection based on BLUP compared to using phenotypes. Preselection based on ssGBLUP yielded similar final accuracy as BLUP for RFI, but a lower accuracy for gain. For the 20:60% preselection intensity, preselection based on BLUP yielded similar final accuracies as preselection based on phenotypes for body weight (0.84 vs. 0.84), RFI (0.88 vs. 0.86) and Gain (0.88 vs. 0.87) while preselection based on ssGBLUP tended to result in the lowest GEBV accuracy at final selection for gain (0.84) and body weight (0.80).

**TABLE 4:** Final selection GEBV accuracy per trait for each preselection scenario averaged over the last 11 selection rounds and the 15 replicates.

			Body weight		RFI		Gain	
Criteria	Preselection % ♂♀	Abbreviation	Accuracy	SE	Accuracy	SE	Accuracy	SE
Phenotype	10:30	P10	0.80	0.002	0.82	0.002	0.81	0.002
BLUP	10:30	B10	0.80	0.003	0.84	0.001	0.82	0.002
ssGBLUP	10:30	G10	0.79	0.002	0.86	0.003	0.82	0.003
Phenotype	15:45	P15	0.81	0.001	0.84	0.002	0.84	0.001
BLUP	15:45	B15	0.81	0.002	0.88	0.002	0.87	0.002
ssGBLUP	15:45	G15	0.82	0.003	0.88	0.002	0.84	0.003
Phenotype	20:60	P20	0.84	0.002	0.86	0.002	0.87	0.001
BLUP	20:60	B20	0.84	0.002	0.88	0.002	0.88	0.001
ssGBLUP	20:60	G20	0.80	0.002	0.87	0.003	0.84	0.001

### 3.3 Family Representation

An overview of the family representation at preselection and final selection is shown in Table 5. At preselection, all families are represented in all preselection strategies, so the number is 600. At final selection, the preselection criteria will reduce the number of families represented. The family representation at final selection is highest for the phenotypic preselection, followed by BLUP and ssGBLUP across all selection intensities. As expected, the number of families represented at final selection goes up for all preselection criteria when the selection intensity goes down. The increments of number of families represented is highest in the phenotypic preselection criteria and mostly profound on the male side. The difference in the number of families selected at preselection diminishes between phenotypic, BLUP and ssGBLUP preselection as the number of birds selected goes up, whereas the difference between the family representation between the BLUP and ssGBLUP criteria remains constant.

**TABLE 5:** Family representation of each selection criteria at final selection.

Preselection strategy	Number of families represented at preselection		Number of families represented at final selection	
	Male	Female	Male	Female
P10	600	600	296	518
B10	600	600	124	288
G10	600	600	116	258
P15	600	600	377	573
B15	600	600	168	384
G15	600	600	160	348
P20	600	600	440	592
B20	600	600	218	465
G20	600	600	203	435

### 3.4 Dispersion Bias

The magnitude of the dispersion bias is depicted in Table 6 for each trait, each preselection intensity and each preselection criteria. Table 6 shows that all Body Weight GEBVs are inflated when only a small proportion of animals is preselected but the inflation is more severe for P10 compared to B10 and G10 (0.83 vs. 0.96 vs. 0.94 respectively). This magnitude of bias for phenotypic selection decreases as the preselection intensity decreases to P15 (0.92) and almost disappears when P20 is used (0.97). Final GEBV following BLUP and ssGBLUP preselection had RCs very close to 1.00 for body weight when 15:45% or more of the animals were preselected. For both RFI and Gain, an RC between 0.95 and 1.02 was obtained, where the lowest values were found for phenotypic and BLUP preselection.

**TABLE 6:** Final GEBV dispersion bias (regression coefficients (RC)) per trait for each preselection scenario at final selection averaged across the last 11 selection rounds and the 15 replicates.

			Body weight		RFI		Gain	
Criteria	Preselection % ♂♀	Abbreviation	RC	SE	RC	SE	RC	SE
Phenotype	10:30	P10	0.81	0.011	1.01	0.003	0.95	0.004
BLUP	10:30	B10	0.95	0.006	0.95	0.005	0.98	0.005
ssGBLUP	10:30	G10	0.93	0.005	0.97	0.005	1.01	0.005
Phenotype	15:45	P15	0.92	0.012	0.98	0.004	1.00	0.003
BLUP	15:45	B15	0.99	0.005	1.02	0.003	1.00	0.003
ssGBLUP	15:45	G15	1.02	0.006	1.01	0.006	1.00	0.003
Phenotype	20:60	P20	0.97	0.007	0.95	0.004	0.99	0.002
BLUP	20:60	B20	0.99	0.005	0.98	0.002	0.98	0.003
ssGBLUP	20:60	G20	1.00	0.004	1.02	0.003	0.97	0.003

### 3.5 Genetic Progress

**FIGURE 3:**
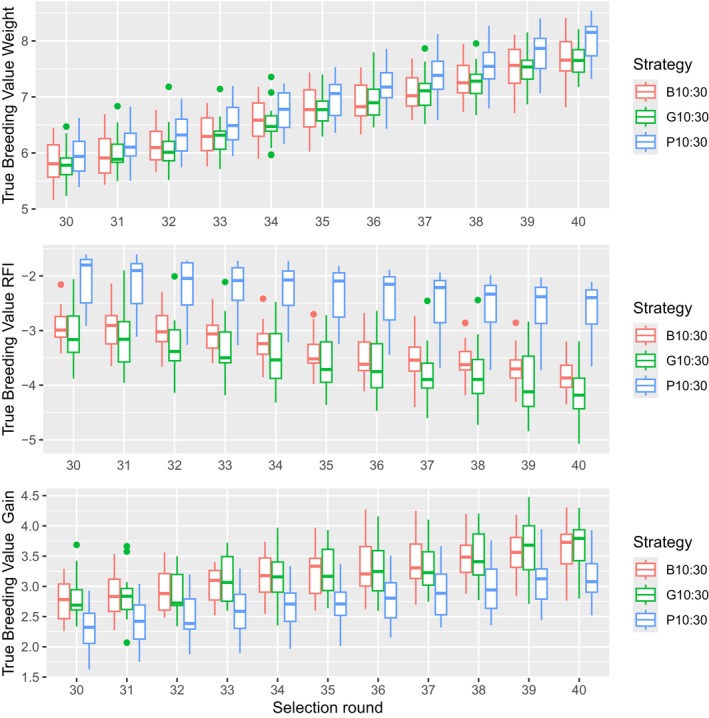
Standardised genetic progress for body weight, RFI and gain at the 10:30% preselection intensity for each strategy. [Colour figure can be viewed at wileyonlinelibrary.com]

The standardised genetic progress, expressed in genetic SD, for each trait for the highest preselection intensity is shown in Figure 3 and represented in Table 7. Figure 3 shows that, when a small proportion of birds is preselected based on phenotype (P10), this results in a significantly higher genetic progress in body weight compared to B10 and G10 at selection round 40, but it shows significantly lower progress in both RFI and Gain. As a result, the total index at selection round 40 is significantly lower compared to B10 and G10 as is seen in Table 7.

**TABLE 7:** The average TBVs for the three traits and the selection index for final selection rounds 30 and 40 (expressed in genetic SD) and the genetic progress computed as the difference (*∆*) between the average TBVs of those two selection rounds.

	TBV body weight			TBV RFI			TBV gain			Total index		
Strategy	Round 30	Round 40	*∆*	Round 30	Round 40	*∆*	Round 30	Round 40	*∆*	Round 30	Round 40	*∆*
P10	5.93^a^	8.00^a^	2.07^a^	−2.04^b^	−2.60^b^	−0.56^b^	2.32^b^	3.16^b^	0.84^a^	8.78^b^	11.73^b^	2.95^b^
B10	5.84^a^	7.69^b^	1.85^b^	−2.93^a^	−3.82^a^	−0.89^a^	2.74^a^	3.64^a^	0.90^a^	9.62^a^	12.66^a^	3.04^a^
G10	5.79^a^	7.65^b^	1.86^b^	−3.09^a^	−4.12^a^	−1.03^a^	2.81^a^	3.68^a^	0.87^a^	9.75^a^	12.89^a^	3.14^a^
P15	5.82^a^	7.86^a^	2.04^a^	−2.57^b^	−3.41^b^	−0.84^b^	2.53^b^	3.37^b^	0.84^a^	9.21^b^	12.36^b^	3.15^a^
B15	5.70^a^	7.61^ab^	1.91^ab^	−3.36^a^	−4.28^a^	−0.92^b^	2.77^ab^	3.64^ab^	0.87^a^	9.85^a^	12.96^a^	3.11^a^
G15	5.63^a^	7.47^b^	1.84^b^	−3.35^a^	−4.44^a^	−1.09^a^	3.03^a^	3.92^a^	0.89^a^	9.91^a^	13.10^a^	3.18^a^
P20	5.71^a^	7.66^a^	1.94^a^	−2.85^b^	−3.81^b^	−0.96^a^	2.69^b^	3.61^b^	0.92^a^	9.42^b^	12.61^b^	3.19^a^
B20	5.65^a^	7.59^a^	1.94^a^	−3.27^a^	−4.23^a^	−0.96^a^	2.86^ab^	3.84^ab^	0.98^a^	9.77^a^	13.00^a^	3.23^a^
G20	5.61^a^	7.49^a^	1.88^a^	−3.28^a^	−4.31^a^	−1.03^a^	3.08^a^	4.06^a^	0.98^a^	9.87^a^	13.09^a^	3.22^a^

*Note:* Within each column, different superscripts (a, b) denote significant differences between strategies within the same preselection intensity.

**FIGURE 4:**
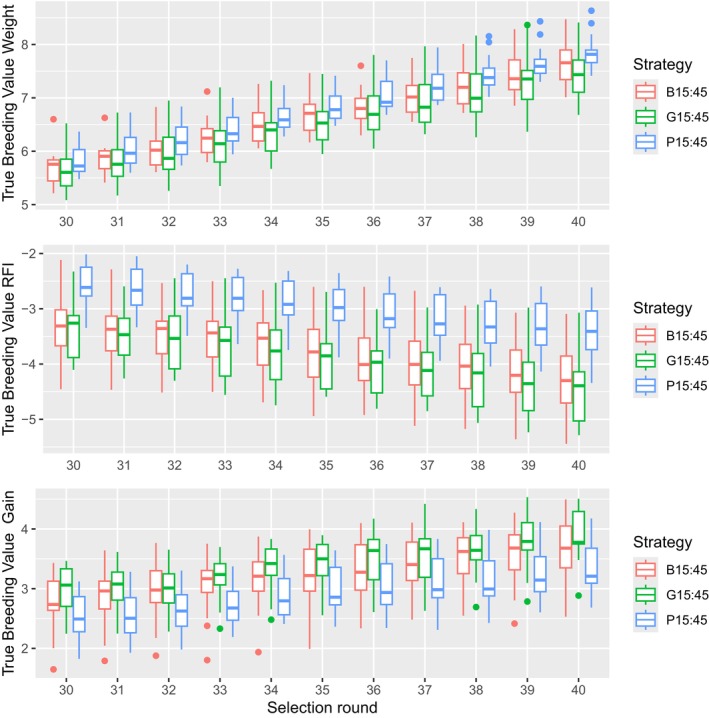
Standardised genetic progress for body weight, RFI and gain at the 15:45% preselection intensity for each strategy. [Colour figure can be viewed at wileyonlinelibrary.com]

**FIGURE 5:**
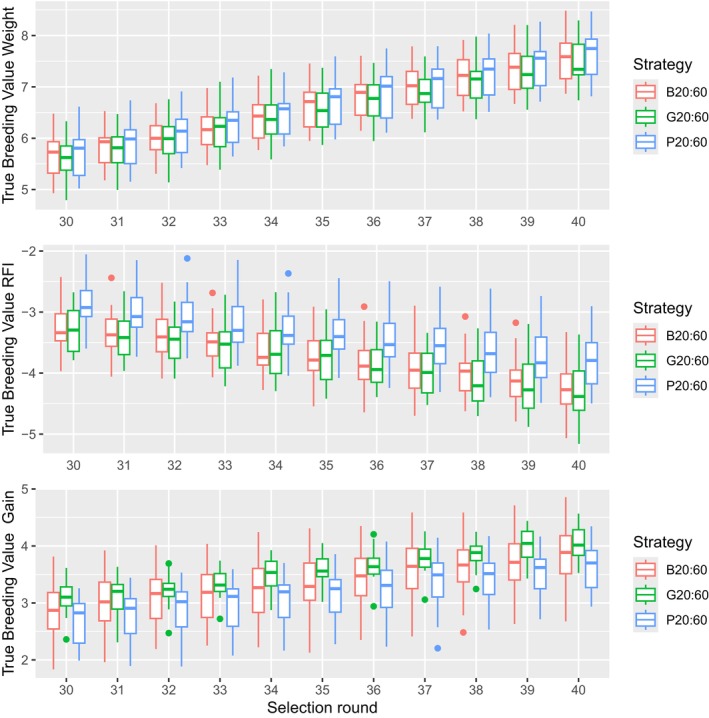
Standardised genetic progress for body weight, RFI and gain at the 20:60% preselection intensity for each strategy. [Colour figure can be viewed at wileyonlinelibrary.com]

When the proportion of preselected birds increased from 10:30% (Figure 3) to 15:45% (Figure 4), the superiority of phenotypic preselection for body weight is reduced but we still see a statistically lower genetic progress in both RFI and Gain compared to B15 and G15 (Table 7). As a result, the index for P15 at selection round 40 is still significantly lower compared to B15 and G15. From Figure 5, it can be seen that the difference in genetic progress between preselection criteria for each trait decreased further when the preselected proportion increased to 20:60%. All preselection criteria result in similar genetic progress for body weight, but P20 is still behind for both RFI and Gain.

Thus, there is a significant disadvantage of using phenotypic preselection on the genetic progress of gain and RFI across all selection intensities. However, similar genetic progress is realised for all traits with preselection based on BLUP or ssGBLUP for each selection intensity.

**FIGURE 6:**
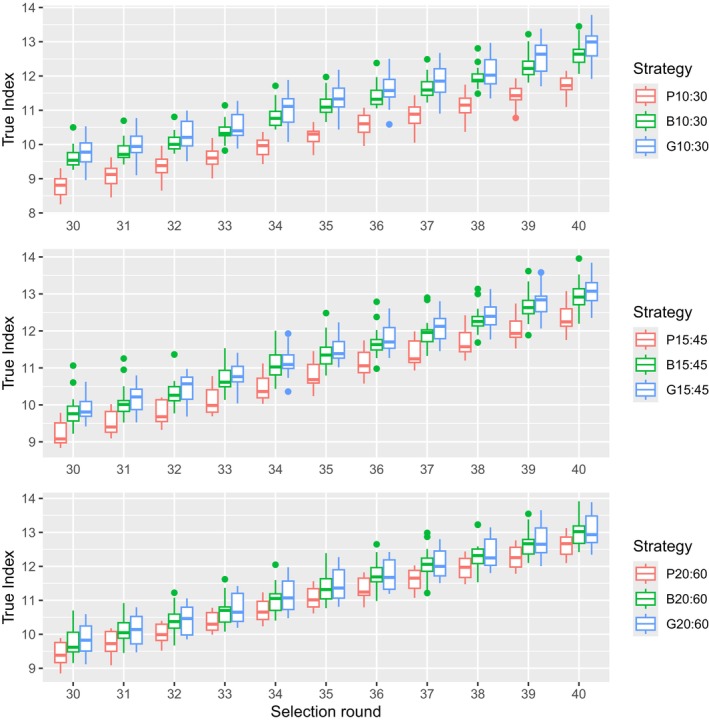
Genetic progress for the selection index for all the preselection intensities for each strategy. [Colour figure can be viewed at wileyonlinelibrary.com]

Figure 6 shows the difference in progress based on the standardised selection index for each criteria for selection round 30–40. It is clear that preselecting on phenotypes will significantly reduce the overall index across all preselection intensities compared to the preselection based on BLUP or ssGBLUP. At the higher selection intensities, G10 outperforms B10 numerically; however, this difference in index is not significant. Interestingly, Table 7 shows that the difference in index between selection round 30 and 40 for selection intensity 15:45% and 20:60% is numerically similar for all preselection criteria, implying that the genetic progress achieved across those 10 rounds is similar, albeit that the selection index values were lower for phenotypic preselection.

Table 8 shows the actual difference in genetic progress computed from TBVs and GEBVs based on all breeder candidates at final selection between selection round 30 and 40. As the regression coefficients showed, the genetic progress computed from the GEBVs for all selection criteria and all three traits always overestimated the genetic progress computed from the TBVs. This was especially the case for the higher selection intensities, 10:30% and 15:45%. The difference was most pronounced for the body weight trait when phenotypic preselection was performed, with P10 and P15 overestimating the genetic progress for body weight by 57% and 37% respectively. In general, the ssGBLUP preselection strategy seems to lead to the lowest overestimation for all traits and the overestimation decreases for all traits for all criteria when more animals are preselected.

**TABLE 8:** The error in the estimated genetic progress based on GEBVs at final selection between rounds 30 and 40 (expressed in genetic SD).

	Genetic progress body weight		Genetic progress RFI		Genetic progress gain	
Strategy	*∆* GEBV versus. TBV	% *∆* ^a^	*∆* GEBV versus. TBV	% *∆*	*∆* GEBV versus. TBV	% *∆*
P10	1.19	57	−0.18	32	0.20	24
B10	0.61	33	−0.27	30	0.22	24
G10	0.42	23	−0.27	26	0.18	21
P15	0.76	37	−0.12	14	0.14	17
B15	0.39	20	−0.18	20	0.17	20
G15	0.21	11	−0.17	16	0.15	17
P20	0.54	28	−0.06	6	0.10	11
B20	0.26	13	−0.13	14	0.10	10
G20	0.12	6	−0.15	15	0.09	9

^a^ Computed as: 100% × (genetic progress GEBVs – genetic progress TBVs)/genetic progress TBVs.

## 4 Discussion

The objective of this study was to evaluate the effect of preselection intensity (10:30, 15:45 and 20:60% of the males and females being selected) and criteria (phenotypic body weight (P), Index based on EBVs (B) and Index based on GEBVs(G)) on the accuracy and bias of GEBVs at final selection, and the overall genetic progress for body weight, RFI, (body weight) gain and the selection index.

### 4.1 Preselection Accuracy

The preselection accuracies of Body Weight, RFI and Gain generally had similar values, which agrees with the fact that the traits had similar heritabilities (Table 1). However, there is an improvement in preselection accuracy when using GEBVs at preselection, except for Gain at the 20:60% preselection intensity. Jibrila et al. (2020) found higher accuracies for ssGBLUP preselection compared to selection based on parental averages. Using ssGBLUP at preselection yielded relevant improvements in preselection accuracy by improving the accuracy of the parental averages. These improvements are expected to be even higher if all individuals available at preselection were genotyped, as the model would be able to predict the Mendelian Sampling terms better (Daetwyler et al. 2012; Hayes, Goddard, and Visscher 2009). However, this is not the case in our strategies, as genotypes were only collected after preselection. The improvement in accuracy with lower preselection intensities for RFI and Gain is likely due to the increased amount of information available on RFI and Gain phenotypes. Body Weight did not show the same improvement across intensities, likely because this trait has phenotypes for all animals available no matter what strategy is performed. The fluctuation in the preselection accuracy for Body Weight was not expected and we do not have a clear explanation for this result.

### 4.2 Family Representation

Different preselection criteria resulted in a different number of families being represented at final selection. It is clear that phenotypic selection will result in a larger representation of families compared to BLUP and ssGBLUP preselection strategies. Additionally, the BLUP preselection strategy shows more representation of families than the ssGBLUP strategy. These results are likely a consequence of the higher preselection accuracy of ssGBLUP, which results in favouring better families, which will reduce the number of families represented at final selection.

### 4.3 Final Selection Accuracy

While considerable differences in preselection accuracy were observed between BLUP and GBLUP, this was not the case for accuracy at final selection. The accuracy was significantly higher at final selection compared to preselection, which is a result of the increased number of phenotypes and genotypes. Every preselected individual was genotyped and had information on their own RFI and Gain performance. All these additional data benefitted the accuracy at final selection for all preselection criteria. The GEBVs at final selection are affected to a limited extent by the available data from relatives and other genotyped animals. It was shown in Table 3 that different preselection criteria indeed resulted in a different representation of families and thereby affecting the data structure, which may affect the accuracy at final selection. However, there appears to only be a slight benefit in selection accuracy at final selection from preselecting with ssGBLUP compared to the other criteria for RFI at higher preselection intensities (10:30%). Suggesting that, at high selection intensity, the data structure at final selection for all selection criteria is sufficient to obtain similar accuracies for at least Body Weight and Gain.

The final selection accuracies of both RFI and Gain are slightly improved with increasing preselection proportions. This is most likely caused by the increased number of phenotypes and genotypes available for (G)EBV calculations, provided that Body Weight does not show as big of an improvement with increasing selection proportions, which is the trait where all phenotypes are collected before preselection no matter what preselection strategy is used. In general, phenotypic preselection tended to result in lower final selection accuracy for RFI and Gain, this disadvantage disappeared when the selection intensity lowered to 20:60%, leading to a less stringent preselection on Body Weight. Slightly higher final selection accuracies were seen for the BLUP criteria compared to phenotypic or ssGBLUP preselection when the preselection intensity was 15:45% or lower. Jibrila et al. (2020) also found higher accuracy for subsequent selection if parental average was used at preselection instead of ssGBLUP. This lower accuracy for the ssGBLUP criteria at final selection is likely a consequence of a more stringent truncation at preselection, because the ssGBLUP GEBVs are more accurate and selecting on these leads to a more severe truncation of the available data after preselection. The expected values of a correlation computed from a truncated distribution are known to be increasingly smaller than the correlation in the full distribution with more stringent truncation (Aitkin 1964). Likewise, with more accurate preselection, the variance among the preselected animals is expected to be lower (Bulmer 1971; Gomez‐Raya and Burnside 1990; Van Grevenhof, Van Arendonk, and Bijma 2012). This reduced variance in turn can reduce the computed accuracy of selection at final selection (Gomez‐Raya and Burnside 1990).

### 4.4 Dispersion Bias

From the results in Table 6, it is clear that stringent phenotypic culling results in large dispersion bias in the GEBVs at final selection for Body Weight. If breeding values are inflated, that is, have too much variance as observed in some presented scenarios, then the average breeding value of a selected set of individuals will be inflated as well. Thus, the observed inflation of GEBVs at P10 for Body Weight (0.81) suggests that the estimated genetic trend for Body Weight will be overestimated. This is also shown in Table 8, where the estimated genetic progress for Body Weight at P10 between selection round 30 and 40 computed from the GEBVs is highly overestimated (+57%). It is known from the literature that sampling extreme families, either based on genetic merit or phenotypes, leads to bias (Chu et al. 2020; Pollak, Van der Werf, and Quaas 1984), especially when the preselection intensity is high (Patry and Ducrocq 2011; Togashi et al. 2018). The magnitude of dispersion bias for phenotypic preselection drops when the truncation becomes less severe with P15 and P20, as all RCs approach 1. G10 shows an RC of 0.93 for Body Weight, so there is a small bias detected as well when this strategy is used, but this bias disappears if 15:45% or more birds are preselected. In general, both BLUP and ssGBLUP preselection strategies show little to no bias at final selection across all preselection intensities. Thus, even with high preselection intensity, when the preselected data are not heavily dependent on one trait (Body Weight), the ssGBLUP used at final selection is able to remove most of the preselection bias (Christensen and Lund 2010; Legarra, Aguilar, and Misztal 2009; Misztal, Legarra, and Aguilar 2009).

It was seen from Table 8 that the observed genetic progress computed from GEBVs always overestimated the actual genetic progress computed from the TBVs. This could be due to the fact that the genetic progress was calculated based on the preselected breeder candidates available at final selection. This preselected pool of candidates is expected to be enriched for animals that have an overestimated GEBV, which leads to overestimation of the average GEBV of this preselected group of animals compared to if all birds were to be considered. It was clear that the overestimated was largest for Body Weight if the P10 or P15 strategy was used, this agrees with the higher bias for Body Weight found for both strategies (0.81 and 0.92 respectively). Nevertheless, no reranking occurred between strategies if the genetic progress was computed based on GEBVs or TBVs.

### 4.5 Genetic Progress

Different genetic trends for the different preselection intensities are shown in Figures 3, 4, 5. For the stronger preselection intensity of 10:30%, it is clear that there is a benefit for genetic progress in Body Weight if animals are phenotypically preselected on Body Weight compared to the BLUP and ssGBLUP criteria (2.07, 1.85 and 1.86 Genetic SD respectively). However, with the 10:30% preselection intensity, the genetic progress between selection round 30 and 40 for RFI is much higher for the BLUP or ssGBLUP criteria compared to phenotypic preselection (−0.89, −1.03 and − 0.56 genetic SD respectively) and noticeably higher for Gain (0.90, 0.89 and 0.84 genetic SD respectively). This is due to the fact that phenotypic preselection will truncate the population based on one trait (Body Weight), while the genetic potential of RFI and Gain is only considered through their genetic correlation with Body Weight. As a result, this strategy will generate less genetic progress in both traits as less eligible breeder candidates are available at final selection, where a multi‐trait index is used. This is clearly shown in Figure 6, where the selection index is significantly lower for P10, compared to B10 and G10. This result is likely due to the unfavourable genetic correlation between body weight and RFI. This aligns with the general knowledge that multi‐trait selection based on a selection index is particularly important for breeding goals with antagonistic genetic correlations among the traits.

When more animals are preselected, that is, 15:45% and 20:60%, the advantage in genetic progress for Body Weight for the phenotypic criteria disappears and the disadvantage for RFI and Gain decreases. However, the lower genetic progress for both RFI and Gain is still significant. Thus, this strategy results in a significantly lower index at selection round 40 across all intensities (Table 7). So, even with more animals being available at final selection, phenotypic preselection is not advised. When we compare BLUP and ssGBLUP preselection strategies, it seems that BLUP does slightly better with genetic progress for Body Weight, while ssGBLUP has a small advantage for the genetic progress of both RFI and Gain at 10:30% and 15:45% preselection intensities; however, this difference is not significant. As a result, there is a small but no significant difference in index favouring ssGBLUP over BLUP preselection, as is seen in Table 7. However, these results could become larger if the genetic parameters or economic index of the study would change. Since the accuracy at final selection did not differ for ssGBLUP and BLUP, the small differences in genetic progress are due to having more accurate preselection, resulting in a better pool of breeder candidates available at final selection for the breeding goal, which is in accordance with the results of Jibrila et al. (2020). Thus, preselection accuracy has a bigger impact than the final selection accuracy and with higher selection intensities ssGBLUP should be considered for preselection. When selected proportions increase, the difference in genetic progress between ssGBLUP and BLUP disappears, as is seen in Figure 6. The more breeder candidates are eligible at final selection, the chances of having similar breeder candidates available at final selection increase, and thus, the preselection accuracy has less of an impact.

In general, the genetic progress is higher when more animals are preselected which was also found by other studies (Jibrila et al. 2020; Martinez et al. 2006; Schrooten et al. 2005). An intuitive explanation is that the chance of culling potentially superior birds at preselection is reduced, while with increasing proportions of birds kept, there is an increase in availability on phenotypic and genotype information. The extra available information for RFI and Gain increases final selection accuracy and thereby genetic progress for those traits. These results may suggest that it is beneficial to preselect more animals, as more genetic progress is made. This additional genetic progress should, however, be weighted against the additional costs for genotyping more animals, as well as measuring RFI and Gain on more animals. It is difficult to weight the additional benefits from the increased genetic progress against the additional costs. Given that going from 10:30% to 15:45% or 20:60% preselection intensity will increase genotyping costs by 50 or 100%, respectively, it seems unlikely that the observed relatively small increase in genetic progress would justify these additional costs.

The improvement in genetic progress by using BLUP or ssGBLUP at preselection is mostly noticed for RFI and Gain, the traits that are not recorded on all breeder candidates placed at hatch but only on the preselected birds. Thus, for a trait that is only measured later in life (i.e., reproduction traits) or only recorded on siblings (i.e., dissection traits), we may expect larger differences in the rate of progress between using BLUP and ssGBLUP at preselection. Also, traits with lower heritability than for the traits considered in our study may benefit more from the use of ssGBLUP at preselection instead of BLUP (Jibrila et al. 2020). Finally, in a scenario where all birds are genotyped at hatch, it is expected that the preselection based on an ssGBLUP index would be even more accurate compared to preselection based on a BLUP index, because this would even further improve the preselection accuracy and thereby increase genetic progress.

## 5 Conclusion

Using ssGBLUP at preselection has a small advantage for the genetic progress when the preselection intensity is high. The benefit of using ssGBLUP is driven by having more accurate breeding values at preselection. When the selection intensity lowers, the benefit of using ssGBLUP over BLUP at preselection gradually disappears. Both BLUP and ssGBLUP did outperform phenotypic preselection.

## Author Contributions

C.A.H. analysed the data and wrote the manuscript. V.B.F., F.B.L. and M.P.L.C. supervised the study and assisted with the interpretation of results and writing of the manuscript. T.T.C. and D.M. have helped C.A.H. with the simulations in ADAM. T.T.C. has shared his QMSim simulation for the foundation population and its haplotypes which were used in these simulations of this study.

## Ethics Statement

The study was done on simulated data.

## Conflicts of Interest

The authors declare no conflicts of interest.

## Supporting information


Figure S1.

## Data Availability

The data used in this study were based on a broiler breeding programme and simulated by ADAM and QMSIM.
