# Supplementary material for: The Effect of Preselection on the Level of Bias and Accuracy in a Broiler Breeder Population, a Simulation Study
Source: J Anim Breed Genet. 2024 Nov 21;142(4):392–407. doi: 10.1111/jbg.12908 (PMC12149501; doi:10.1111/jbg.12908)
Supplement: Supplementary file 1 — Figure S1. [file JBG-142-392-s001.docx]

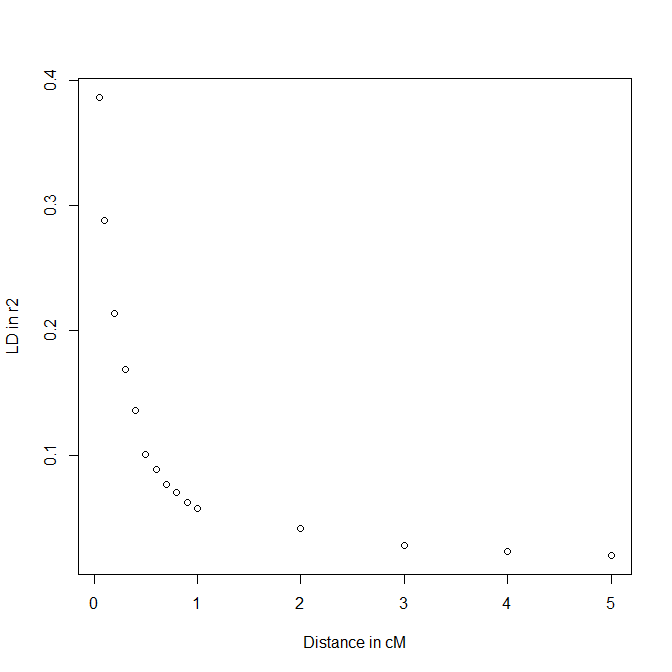


**Supplementary Figure S1.** Decay of (average) LD between loci with increasingly larger distances between them.
